# Effects of feeding high-protein corn distillers dried grains and a mycotoxin mitigation additive on growth performance, carcass characteristics, and pork fat quality of growing–finishing pigs^1^

**DOI:** 10.1093/tas/txaa051

**Published:** 2020-05-04

**Authors:** Zhaohui Yang, Pedro E Urriola, Adrienne Hilbrands, Lee J Johnston, Gerald C Shurson

**Affiliations:** 1 Department of Animal Science, University of Minnesota, St. Paul, MN; 2 West Central Research and Outreach Center, University of Minnesota, Morris, MN

**Keywords:** carcass characteristics, growing, finishing pigs, growth performance, high-protein distillers dried grains, mycotoxins, pork fat quality

## Abstract

Two experiments investigated the effects of feeding diets containing 30% of novel high-protein distillers dried grains (HP-DDG) sources to growing–finishing pigs on growth performance, carcass characteristics, and pork fat quality. A four-phase feeding program was used in both experiments, and diets within phases were formulated based on National Research Council (NRC; 2012) recommendations for metabolizable energy and standardized ileal digestible amino acid content of HP-DDG. In Exp. 1, a total of 144 pigs (body weight [BW] = 20.3 ± 1.6 kg) were fed either corn-soybean meal control diets (CON) or 30% HP-DDG diets (HP-DDG) containing 0.7 mg/kg deoxynivalenol (DON), 0.1 mg/kg fumonisins (FUM), and 56 μg/kg zearalenone (ZEA) for 8 wk. On week 9, a mycotoxin mitigation additive (MA) was added to CON and HP-DDG diets, resulting in a 2 × 2 factorial arrangement of treatments consisting of: CON, CON + MA, HP-DDG, and HP-DDG + MA. Pigs fed HP-DDG had lower (*P* < 0.01) average daily gain (ADG) and average daily feed intake (ADFI) compared with those fed CON during the first 8 wk. After MA was added to diets, pigs fed HP-DDG diets without MA had lower (*P* < 0.05) overall ADG than those fed HP-DDG + MA and less (*P* < 0.05) final BW than pigs fed CON or CON + MA. Adding MA to HP-DDG diets containing relatively low concentrations of mycotoxins was effective in restoring growth performance comparable to feeding CON. In Exp. 2, a different source of HP-DDG was used, and mycotoxin MAs were added to all diets at the beginning of the trial. A total of 144 pigs (BW = 22.7 ± 2.3 kg) were fed either a corn-soybean meal control diet or a 30% HP-DDG diet containing 0.5 mg/kg DON and 0.8 mg/kg FUM for 16 wk. Pigs fed HP-DDG diets had less (*P* < 0.01) final BW and ADG than pigs fed CON, but there were no differences in ADFI. Feeding the HP-DDG diets reduced (*P* < 0.01) hot carcass weight, carcass yield, longissimus muscle area (LMA), and percentage of carcass fat-free lean compared with pigs fed CON but did not affect backfat (BF) depth. Pigs fed HP-DDG had less (*P* < 0.01) saturated fatty acid (SFA) and monounsaturated fatty acid (MUFA) content and greater (*P* < 0.01) polyunsaturated fatty acid (PUFA) and iodine value in BF than pigs fed CON. These results suggest that feeding diets containing relatively low concentrations of co-occurring mycotoxins can be detrimental to growth performance, and the addition of MA alleviated the growth reduction. Feeding 30% HP-DDG reduced BW, ADG, carcass yield, LMA, and percentage of fat-free lean of growing–finishing pigs but yielded acceptable pork fat quality.

## INTRODUCTION

Previous studies (Widmer e al., 2008; Kim et al., 2009; Jacela et al., 2010) have measured metabolizable energy (ME) and standardized ileal digestible (SID) amino acid (AA) content in high-protein distillers dried grains (HP-DDG) produced using the old front-end fractionation processes, and these data were used in developing nutrient composition tables in NRC (2012). These nutritional composition values appeared to be accurate for previous HP-DDG sources because studies have shown that adding up to 30% of HP-DDG to growing–finishing diets had no effect on overall growth performance or carcass characteristics (Widmer et al., 2008; Kim et al., 2009; Gutierrez et al., 2014). However, after these earlier studies, new technologies have been implemented by some U.S. ethanol plants to produce HP-DDG with substantially different nutritional composition. For example, new HP-DDG sources contain less crude protein (CP; 37–39%) and greater ether extract (EE; 7–10%) and phosphorus (0.6–0.7%) compared with previous HP-DDG sources (45%, 3.5%, and 0.36%, respectively) reported by NRC (2012), as well as different AA profiles. Because there is limited information on the ME and SID AA content in these novel HP-DDG sources, it is unknown if formulating swine diets using the ME and SID AA values from NRC (2012) in diet formulation are still appropriate for achieving optimal growth performance and carcass composition.

Corn is susceptible to mold growth and subsequent mycotoxin production when subjected to stressful environmental conditions during production, harvest, and storage. When mycotoxin-contaminated corn is used to produce ethanol and coproducts, the mycotoxin concentrations are increased by about 3-fold in coproducts (Schaafsma et al., 2009). Recent surveys have reported a high prevalence of natural co-occurrence of mycotoxins in U.S. corn (Hendel et al., 2017). Adverse health and growth performance effects of mycotoxins have been observed when pigs have been fed diets containing mycotoxin concentrations that have generally been considered safe (Alizadeh et al., 2015). Therefore, it is possible that feeding diets containing multiple mycotoxins at low concentrations could have interactive and additive effects on reducing growth and health of pigs. Previous studies (Patience et al., 2014; Frobose et al., 2015; Thanh et al., 2015) have shown that the addition of a specific mycotoxin mitigation additive (MA) to mycotoxin-contaminated swine diets can be effective in alleviating negative effects on growth performance. Thus, the addition of MA may minimize the confounding effects of mycotoxins on growth performance responses when evaluating the nutritional value of new HP-DDG sources for growing–finishing pigs. Therefore, we hypothesized that formulating diets containing 30% of novel HP-DDG sources, with low concentrations of mycotoxins, and using NRC (2012) nutritional values would result in acceptable growth performance, carcass characteristics, and pork fat quality with the addition of MA.

## MATERIALS AND METHODS

The experimental protocols used in this study were approved by the University of Minnesota Institutional Animal Care and Use Committee (protocol # 1501-32226A and 1607-34026A).

### HP-DDG Sources

Sources of HP-DDG were obtained from IGPC Ethanol, Inc. (Aylmer, Ontario, Canada; Exp. 1) and ICM Inc., (St. Joseph, MO; Exp. 2) and delivered to the University of Minnesota West Central Research and Outreach Center (WCROC) in Morris, MN. Upon arrival, samples of HP-DDG (2 kg) were collected and sent to Midwest Laboratories (Omaha, NE) for chemical analysis and mycotoxin determination. Analyses were performed using Association of Official Analytical Chemists (AOAC; 2012) procedures for moisture (method 930.15), EE (method 945.16), CP (method 990.03), neutral detergent fiber (NDF; method 2001.11), AA profile (method 994.12 Alt III), Ca and P (method 985.01), and mycotoxins (method 2008.02). Relatively low concentrations of deoxynivalenol (DON; 1.7 mg/kg), total fumonisins (FUM; 0.60 mg/kg), and zearalenone (ZEA; 0.2 mg/kg) were present in the HP-DDG source used in Exp. 1 (Table 1). Similarly, relatively low concentrations of DON (1.0 mg/kg), FUM (3.80 mg/kg), and ZEA (0.06 mg/kg) were present in the HP-DDG source used in Exp. 2 (Table 1). Based on the Food and Drug Administration (FDA) Mycotoxin Regulatory Guidance for advisory levels of DON and FUM in grain byproducts fed to swine, 5 mg/kg is recommended as the maximum concentration of DON (not to exceed 20% of the diet), and no more than 20 mg/kg FUM (not to exceed 50% of the diet) is recommended for corn byproducts (National Grain and Feed Association [NGFA], 2019). There are no FDA guidelines for ZEA. The HP-DDG sources contained less than the maximum recommended guidelines for DON and FUM, and it was assumed that there would be minimal, if any, effect on growth performance from adding 30% of these HP-DDG sources to grower–finisher swine diets in this study.

**Table 1.** Analyzed nutrient and mycotoxin composition of HP-DDG sources used in Exp. 1 and Exp. 2 compared with NRC (2012) values (as-fed basis)

Item	Exp. 1	Exp. 2	NRC (2012)
DM, %	89.0	94.0	91.2
CP, %	37.10	38.6	45.4
EE, %	7.59	10.1	3.5
NDF, %	33.0	36.0	33.6
Ca, %	0.01	0.07	0.02
P, %	0.64	0.71	0.36
ME, kcal/kg	ND	ND	3,732
Indispensable AA^*a*^ %			
Arg	1.80	1.69	1.62 (85)
His	1.15	1.01	1.07 (79)
Ile	1.48	1.43	1.83 (80)
Leu	4.40	3.95	6.18 (86)
Lys	1.29	1.14	1.22 (69)
Lys: CP × 100	3.48	2.95	2.69
Met	0.72	1.09	0.93 (86)
Phe	1.90	1.89	2.42 (84)
Thr	1.46	1.31	1.59 (75)
Trp	0.36	0.35	0.24 (82)
Val	2.51	1.94	2.12 (78)
Mycotoxins			
DON, mg/kg	1.70	1.00	ND
Total FUM, mg/kg	0.60	3.80	ND
ZEA, mg/kg	0.20	0.06	ND

ND, no data available.

^*a*^Numbers in parentheses are SID coefficients from NRC (2012).

### Diet Formulation and Analysis

A four-phase feeding program, based on body weight (BW) of pigs (20–50 kg, 50–75 kg, 75–100 kg, and 100–120 kg), was used in both experiments. Diets were formulated on an SID AA and standardized total tract digestibility (STTD) phosphorus basis using a 5% safety margin above the NRC (2012) requirements to provide similar SID Lysine:ME (Lys:ME) among diets within each phase. The ME content for HP-DDG from NRC (2012) was used in formulating experimental diets, while SID AA coefficients and the STTD of P coefficient for HP-DDG were obtained from NRC (2012) and used with the analyzed chemical composition of each HP-DDG source to calculate SID AA content and STTD P content. Metabolizable energy, SID AA, and STTD of P values for corn and soybean meal were obtained from NRC (2012) and used in diet formulation. Diet changes were made when the average pig BW of each pen was within 3 kg of the target beginning BW for each subsequent phase. Samples of complete diets were obtained after mixing, frozen at −20 °C, and analyzed for nutrient composition at the University of Missouri Agricultural Experiment Station Chemical Laboratory (AESCL; Columbia, MO) using the following AOAC (2012) procedures for dry matter (method 934.01), CP (method 984.13), EE (method 920.39), ash (method 942.05), and AA profile (method 982.30). Mycotoxin content of the experimental diets was determined at North Dakota State University Veterinary Diagnostic Laboratory (Fargo, ND), where mycotoxins were extracted in acetonitrile water followed by liquid chromatography/mass spectrometry/mass spectrometry detection (Varga et al., 2012).

### Experimental Design and Dietary Treatments

In Exp. 1, a total of 144 pigs (initial BW = 20.3 ± 1.6 kg) were used in a randomized complete block design for a 16-wk feeding period. Pigs were blocked by initial BW, equalized across treatments for sex, and assigned to pens (9 pigs per pen). Pens within blocks were assigned randomly to one of two dietary treatments. Dietary treatments during the first 8 wk consisted of either a corn-soybean meal control diet (CON) or a 30% HP-DDG diet (HP-DDG). Due to consistent reductions in average daily feed intake (ADFI) and average daily gain (ADG) observed when feeding the HP-DDG diets during the initial 8-wk of the study, we suspected that mycotoxin contamination was a likely cause of the observed reduction in feed intake and growth rate. Therefore, at the end of week 8, the experiment was re-designed as a 2 × 2 factorial arrangement of treatments where 4 pens of pigs fed CON and 4 pens of pigs fed HP-DDG diets were selected randomly to be fed their respective diets containing 0.25% of a commercial mycotoxin MA (Defusion Plus, Provimi, Lewisburg, OH), resulting in 4 pens of each dietary group: CON, CON + 0.25% MA (CON + MA), 30% HP-DDG, and 30% HP-DDG + 0.25% MA (HP-DDG + MA; Tables 2 and 3). This mycotoxin MA was chosen because of its demonstrated effectiveness in preventing reductions in ADFI and ADG when feeding DON-contaminated diets to nursery and growing–finishing pigs in previous studies (Patience et al., 2014; Frobose et al., 2015; Thanh et al., 2015). These new diets were fed for the remaining 8 wk of the 16-wk feeding period.

**Table 2.** Diet and calculated nutrient composition for phase 1 and 2 diets fed for the first 8 wk in Exp. 1 (as-fed basis)^*a*^

	Phase 1 (20–50 kg BW)		Phase 2 (50–75 kg BW)	
Item	CON	HP-DDG	CON	HP-DDG
Ingredients, %				
Corn	64.37	56.77	71.46	61.80
Soybean meal (47%)	33.80	10.50	26.80	5.91
HP-DDG	–	30.00	–	30.00
CON basemix^*b*^	1.83	–	1.74	–
HP basemix^*b*^	–	2.23	–	1.87
Total	100.00	100.00	100.00	100.00
Calculated composition				
ME, kcal/kg	3,300	3,395	3,310	3,410
CP, %	21.44	20.82	18.68	19.04
EE, %	2.75	4.41	2.89	4.52
NDF, %	8.64	15.94	8.71	16.02
Ca, %	0.70	0.78	0.65	0.68
Total P, %	0.60	0.56	0.57	0.54
STTD P,^*c*^ %	0.33	0.35	0.31	0.33
SID AA,^*d*^ %				
Lys	1.01	1.04	0.84	0.86
Met	0.30	0.33	0.27	0.31
Thr	0.67	0.66	0.58	0.60
Trp	0.23	0.18	0.20	0.15
Leu	1.61	1.94	1.45	1.84
Ile	0.79	0.69	0.67	0.61
Val	0.86	0.97	0.74	0.89
SID Lys/ME, g/Mcal	3.06	3.06	2.53	2.53
Analyzed composition				
CP, %	18.28	19.88	16.91	18.05
NDF, %	7.59	13.72	7.98	13.21
Lys, %	1.11	1.24	1.00	1.04
Met, %	0.28	0.38	0.27	0.35
Thr, %	0.75	0.81	0.68	0.71
Trp, %	0.25	0.20	0.22	0.17

^*a*^Phase 1 CON diet contained 0.1 mg/kg DON; Phase 1 HP-DDG diet contained 0.7 mg/kg DON, 0.1 mg/kg FUM, and 56 μg/kg ZEA.

^*b*^CON basemix contained 50.9% limestone, 10.9% monocalcium phosphate, 1.2% soybean oil, 21.8% salt, 13.9% VTM premix, and 1.2% phyzyme 600 (Danisco Animal Nutrition, St. Louis, MO); HP basemix contained 17.60% L-lysine HCl, 66.3% limestone, 1.1% soybean oil, 18.0% salt, 13.5% VTM premix, and 1.1% phyzyme 600. VTM premix = vitamin–mineral premix, which contained the following nutrients per kilogram of premix: 3,527,200 IU vitamin A, 661,600 IU vitamin D_3_, 13,200 IU vitamin E, 1,320 mg vitamin K, 2,200 mg riboflavin, 13,240 mg niacin, 8,800 mg pantothenic acid, 12.0 mg vitamin B_12_, 120 mg iodine as ethylenediamine dihydroiodide, 120 mg selenium as sodium selenite, 22,040 mg zinc as zinc oxide, 13,240 mg iron as ferrous sulfate, 2,200 mg manganese as manganous oxide, and 1,560 mg copper as copper sulfate.

^*c*^ NRC (2012) recommended STTD coefficients were used for corn, soybean meal, and HP-DDG.

^*d*^ NRC (2012) recommended SID coefficients were used for corn, soybean meal, and HP-DDG.

**Table 3.** Diet and calculated nutrient composition for phase 3 and 4 with or without mycotoxin MA fed during the last 8 wk in Exp. 1 (as-fed basis)^*a*^

	Phase 3 (75–100 kg BW)				Phase 4 (100–120 kg BW)			
	CON		HP-DDG		CON		HP-DDG	
Item	−MA	+MA	−MA	+MA	−MA	+MA	−MA	+MA
Ingredients, %								
Corn	76.37	76.12	66.22	65.97	80.14	79.89	68.19	67.94
Soybean meal (47%)	21.98	21.98	1.60	1.60	18.40	18.40	–	–
HP-DDG	–	–	30.00	30.00	–	–	30.00	30.00
CON basemix^*b*^	1.65	1.65	–	–	1.46	1.46	–	–
HP basemix^*b*^	–	–	1.78	1.78	–	–	1.48	1.48
Defusion Plus^*c*^	–	0.25	–	0.25	–	0.25	–	0.25
Total	100.00	100.00	100.00	100.00	100.00	100.00	100.00	100.00
Calculated composition								
ME, kcal/kg	3,318	3,310	3,422	3,414	3,328	3,320	3,436	3,428
CP, %	16.79	16.76	17.35	17.33	15.39	15.37	16.75	16.73
EE, %	2.99	2.99	4.61	4.61	3.07	3.07	4.65	4.65
NDF, %	8.76	8.76	16.07	16.07	8.81	8.81	16.11	16.11
Total Ca, %	0.62	0.62	0.65	0.65	0.57	0.57	0.57	0.57
Total P, %	0.54	0.54	0.53	0.52	0.52	0.52	0.52	0.51
STTD P,^*d*^ %	0.30	0.30	0.32	0.32	0.28	0.28	0.32	0.32
SID AA,^*e*^ %								
Lys	0.72	0.72	0.74	0.74	0.63	0.63	0.65	0.65
Met	0.24	0.24	0.29	0.29	0.23	0.23	0.29	0.29
Thr	0.51	0.51	0.54	0.54	0.46	0.46	0.52	0.52
Trp	0.17	0.17	0.13	0.13	0.15	0.15	0.12	0.12
Leu	1.34	1.34	1.74	1.74	1.26	1.26	1.71	1.71
Ile	0.59	0.59	0.54	0.54	0.53	0.53	0.51	0.51
Val	0.66	0.66	0.82	0.82	0.61	0.61	0.80	0.80
SID Lys:ME, g/Mcal	2.17	2.17	2.17	2.18	1.90	1.91	1.90	1.90
Analyzed composition								
CP, %	16.65	16.31	16.17	16.89	15.06	14.01	14.76	16.41
NDF, %	8.21	8.74	13.71	13.86	8.32	7.69	12.38	14.63
Lys, %	0.78	0.84	0.82	0.95	0.74	0.73	0.85	0.83
Met, %	0.21	0.22	0.30	0.32	0.21	0.22	0.32	0.35
Thr, %	0.55	0.58	0.60	0.64	0.55	0.53	0.62	0.67
Trp, %	0.20	0.16	0.14	0.15	0.18	0.18	0.14	0.15

^*a*^HP-DDG diets contained 0.7 mg/kg DON, 0.1 mg/kg FUM, and 56 μg/kg ZEA, and CON diets contained 0.1 mg/kg DON.

^*b*^CON basemix contained 50.9% limestone, 10.9% monocalcium phosphate, 1.2% soybean oil, 21.8% salt, 13.9% VTM premix, and 1.2% phyzyme 600 (Danisco Animal Nutrition, St. Loius, MO); HP basemix contained 17.60% L-lysine HCl, 66.3% limestone, 1.1% soybean oil, 18.0% salt, 13.5% VTM premix, and 1.1% phyzyme 600. VTM premix = vitamin–mineral premix, which contained the following nutrients per kilogram of premix: 3,527,200 IU vitamin A, 661,600 IU vitamin D_3_, 13,200 IU vitamin E, 1,320 mg vitamin K, 2,200 mg riboflavin, 13,240 mg niacin, 8,800 mg pantothenic acid, 12.0 mg vitamin B_12_, 120 mg iodine as ethylenediamine dihydroiodide, 120 mg selenium as sodium selenite, 22,040 mg zinc as zinc oxide, 13,240 mg iron as ferrous sulfate, 2,200 mg manganese as manganous oxide, and 1,560 mg copper as copper sulfate.

^*c*^Defusion Plus (Provimi, Lewisburg, OH) contains a proprietary blend of preservatives, AAs, antioxidants, and yeast products.

^*d*^ NRC (2012) recommended STTD coefficients were used for corn, soybean meal, and HP-DDG.

^*e*^ NRC (2012) recommended SID coefficients were used for corn, soybean meal, and HP-DDG.

Similar to the HP-DDG source used in Exp. 1, the HP-DDG source used in Exp. 2 was also contaminated with relatively low concentrations of DON, FUM, and ZEA. However, in contrast to the HP-DDG in Exp. 1, the HP-DDG from Exp. 2 contained lower concentrations of DON and ZEA but a greater concentration of FUM. Because of the effectiveness of adding MA to mycotoxin-contaminated diets in Exp. 1, the same MA was incorporated in all experimental diets fed in Exp. 2, along with an additional additive (Defusion 501; Provimi, Lewisburg, OH) based on supplier recommendations.

In Exp. 2, 144 gilts and barrows (initial BW = 22.7 ± 2.3 kg) were grouped by sex, blocked by initial BW, and assigned to pens (nine pigs per pen). Pens within blocks were assigned randomly to one of two dietary treatments in a complete randomized block design. This design resulted in eight pens per dietary treatment and four pens per sex per treatment. Dietary treatments for the entire experiment consisted of feeding either CON or 30% HP-DDG diets (Table 4) for 16 wk.

**Table 4.** Diet composition and calculated nutrient composition in Exp. 2 (as-fed basis)^*a*^

	Phase 1 (25–50 kg)		Phase 2 (50–75 kg)		Phase 3 (75–100 kg)		Phase 4 (100–120 kg)	
Item	CON	HP-DDG	CON	HP-DDG	CON	HP-DDG	CON	HP-DDG
Ingredient, %								
Corn	62.79	51.63	70.79	59.57	75.88	64.29	79.87	67.60
Soybean meal (47%)	35.00	15.30	27.10	7.80	22.10	3.20	18.30	–
HP-DDG	–	30.00	–	30.00	–	30.00	–	30.00
CON basemix^*b*^	1.91	–	1.81	–	1.72	–	1.53	–
HP basemix^*b*^	–	2.77	–	2.33	–	2.21	–	2.10
Defusion additives^*c*^	0.30	0.30	0.30	0.30	0.30	0.30	0.30	0.30
Total	100.0	100.0	100.0	100.0	100.0	100.0	100.0	100.0
Calculated composition								
ME, kcal/kg	3,286	3,379	3,297	3,401	3,306	3,409	3,316	3,416
CP, %	21.88	23.14	18.77	20.21	16.80	18.40	15.32	17.15
EE, %	2.72	5.06	2.88	5.22	2.98	5.32	3.06	5.38
NDF %	8.59	16.76	8.67	16.87	8.73	16.92	8.78	16.96
Total Ca, %	0.70	0.82	0.66	0.70	0.62	0.66	0.57	0.63
Total P, %	0.60	0.60	0.56	0.57	0.54	0.55	0.52	0.53
STTD P,^*d*^ %	0.33	0.37	0.31	0.35	0.30	0.34	0.28	0.34
SID AA,^*e*^ %								
Lys	1.04	1.07	0.84	0.87	0.72	0.74	0.63	0.65
Met	0.30	0.45	0.27	0.42	0.24	0.40	0.23	0.38
Thr	0.69	0.65	0.58	0.55	0.51	0.48	0.46	0.44
Trp	0.24	0.20	0.20	0.16	0.17	0.14	0.15	0.12
Leu	1.64	1.94	1.46	1.77	1.34	1.71	1.25	1.62
Ile	0.81	0.75	0.68	0.63	0.60	0.58	0.53	0.52
Val	0.88	0.91	0.75	0.79	0.67	0.75	0.61	0.69
SID Lys: ME, g/Mcal	3.16	3.17	2.56	2.56	2.19	2.18	1.90	1.90
Analyzed composition								
CP, %	20.82	22.02	19.33	19.71	15.78	17.62	13.52	15.98
NDF, %	8.69	15.95	7.79	15.08	7.28	14.73	7.71	15.92
Lys, %	1.28	1.41	0.98	1.21	0.77	0.96	0.72	0.78
Met, %	0.30	0.38	0.31	0.35	0.20	0.35	0.20	0.34
Thr, %	0.81	0.84	0.78	0.74	0.54	0.69	0.53	0.61
Trp, %	0.26	0.22	0.26	0.19	0.17	0.16	0.15	0.13

^*a*^Phase 1 HP-DDG diet contained 0.5 mg/kg DON, 0.8 mg/kg FUM, and 56 μg/kg ZEA; phase 1 CON diet contained 0.3 mg/kg DON; other diets were not analyzed.

^*b*^CON basemix contained 49.25% limestone, 10.50% monocalcium phosphate, 1.00% soybean oil, 21.00% salt, 13.00% VTM premix and 5.25% phyzyme 600 (Danisco Animal Nutrition, St. Louis, MO); HP basemix contained 17.60% L-lysine HCl, 53.00% limestone, 1.00% soybean oil, 14.00% salt, 10.50% VTM premix, and 3.90% phyzyme 600. VTM premix = vitamin–mineral premix, which contained the following nutrients per kg of premix: 3,527,200 IU vitamin A, 661,600 IU vitamin D_3_, 13,200 IU vitamin E, 1,320 mg vitamin K, 2,200 mg riboflavin, 13,240 mg niacin, 8,800 mg pantothenic acid, 12.0 mg vitamin B_12_, 120 mg iodine as ethylenediamine dihydroiodide, 120 mg selenium as sodium selenite, 22,040 mg zinc as zinc oxide, 13,240 mg iron as ferrous sulfate, 2,200 mg manganese as manganous oxide, and 1,560 mg copper as copper sulfate.

^*c*^Defusion Plus and Defusion 501 (Provimi, Lewisburg, OH) were incorporated at rates of 0.25% and 0.05%, respectively. Defusion Plus contains a proprietary blend of preservatives, AAs, antioxidants, and yeast products; Defusion 501 contains bentonite and mineral oil.

^*d*^ NRC (2012) recommended STTD coefficients were used for corn, soybean meal, and HP-DDG.

^*e*^ NRC (2012) recommended SID coefficients were used for corn, soybean meal, and HP-DDG.

### Animal Housing and Growth Performance Data Collection

Experiment 1 was conducted in the spring of 2016, and Exp. 2 was conducted in the fall of 2016. Both experiments were conducted in an environmentally controlled confinement grower–finisher facility at the University of Minnesota WCROC. Each pen (1.6 × 4.5 m) consisted of completely slatted concrete flooring, a nipple waterer, and a stainless steel self-feeder with four feeding spaces. Feeders and waterers were checked daily and maintained to provide ad libitum access to feed and water. Pigs were observed daily for health to identify any sick or injured pigs, and sick or injured pigs were removed from study immediately. The BW of individual pigs in each pen was recorded at the beginning of the experiments, and once every 2 wk thereafter, to calculate ADG. Feed added and removed from feeders was weighed and recorded to calculate ADFI and gain–feed ratio (G:F) for each pen.

### Carcass Measurements

Carcass measurements were collected from pigs in Exp. 2 but not from pigs in Exp. 1. When the average BW of pens reached 120 kg, real-time ultrasonic measurement (Exago model, Echo Control Medical, Angouleme, France) of backfat (BF) depth and longissimus muscle area (LMA) between the 10th and 11th ribs was performed by a certified technician. Images were digitized and computed using Biosoft Toolbox II for Swine software (version 2.5.0.6; Biotronics, Inc., Ames, IA). After ultrasound measurements were obtained, final BW was determined, and all pigs were individually tattooed and transported approximately 5 h to a commercial abattoir (Hormel Foods, Austin, MN) in one group for harvest and BF sample collection. Hot carcass weights were recorded to calculate carcass yield, and percentage of carcass fat-free lean (FFL%) for each carcass was calculated using National Pork Producers Council (NPPC; 2000) equations. Samples of BF were collected from two pigs per pen with final BW closest to the pen average. All BF samples were collected from the left side of the carcass at the midline opposite the last rib and included all three fat layers. Samples were sent to the University of Missouri AESCL for fatty acid profile analysis (method 996.06; AOAC, 2012) and subsequent calculation of iodine value (IV) using the American Oil Chemists’ Society (AOCS; 1998) published equation: IV= [C16:1] × 0.95 + [C18:1] × 0.86 + [C18:2] × 1.732 + [C18:3] × 2.616 + [C20:1] × 0.785 + [C22:1] × 0.723, where brackets indicate concentration.

### Statistical Analysis

Data were analyzed using the MIXED procedure of SAS 9.4 (SAS Inst. Inc., Cary, NC) as a randomized complete block design. Pen served as the experimental unit and growth performance data were analyzed using repeated measurements in time. For carcass traits and pork fat quality, individual pig was used as experimental unit, and hot carcass weight (HCW) was used as a covariate for carcass yield, BF depth, LMA, and FFL%. In Exp.1, dietary treatment, MA, and diet × MA were included as fixed effects. Because pig BW at week 8 was different after redistributing dietary treatments into four groups, BW at week 8 was used as a covariate in further analysis. In Exp. 2, diet and sex were included as fixed effects. The univariate test was used to evaluate the normality of residuals within the model, as well as test for outliers and equal distribution in variance. Data were reported as least-squares means and were separated by the PDIFF option with a Tukey adjustment. Least squares means comparisons with values of *P* < 0.05 were considered significant, and values with 0.05 < *P* < 0.1 were considered as trends.

## RESULTS AND DISCUSSION

### Nutrient Composition and Mycotoxin Concentrations of HP-DDG and Experimental Diets

The HP-DDG source used in Exp. 1 contained 37.1% CP, 7.6% EE, 0.64% P, and 33% NDF, whereas the HP-DDG source used in Exp. 2 contained 38.6% CP, 10.1% EE, 0.71% P, and 36% NDF (Table 1). The two sources were produced by the same process but at different ethanol facilities, resulting in different chemical composition. Compared with nutrient composition values from NRC (2012) for HP-DDG (45.6% CP, 3.5% EE, 0.36% P, and 33.6% NDF), which represent HP-DDG sources produced by old “front-end” fractionation technology, these new HP-DDG sources had less CP, similar NDF, greater P and EE content, and different AA profiles. This variability in nutrient composition of HP-DDG is similar in magnitude to the variability observed in corn distillers dried grains with solubles (DDGS) (Zeng et al. 2017). Interestingly, the Lys:CP in the HP-DDG products was greater than average Lys:CP in corn DDGS (Zeng et al., 2017) and that of HP-DDG in NRC (2012).

The HP-DDG source used in Exp. 1 contained 1.7 mg/kg DON, 0.6 mg/kg FUM, and 0.2 mg/kg ZEA, whereas HP-DDG in Exp. 2 contained 1.0 mg/kg DON, 3.8 mg/kg FUM, and 62 µg/kg ZEA (Table 1). Phase 1 diets in Exp. 1 were analyzed for mycotoxin concentrations, and the CON diet contained 0.1 mg/kg DON, while the 30% HP-DDG diet contained 0.7 mg/kg DON, 0.1 mg/kg FUM, and 56 μg/kg ZEA, which were consistent with calculated concentrations. Therefore, diets fed during phases 2, 3, and 4 were not analyzed for mycotoxin concentrations. In Exp. 2, phase 1 diets were also analyzed for mycotoxin concentrations, where the CON diet containing 0.3 mg/kg DON and the 30% HP-DDG diet contained 0.5 mg/kg DON and 0.8 mg/kg FUM. These results also indicated that calculated mycotoxin concentrations in diets were consistent with analyzed values, and no further mycotoxin analysis was performed for phases 2, 3, and 4 diets in Exp. 2.

### Exp. 1 Growth Performance

Six pigs (CON = 1, HP-DDG = 3, and HP-DDG + MA = 2) were removed from the experiment for reasons unrelated to dietary treatments. All other pigs remained in the 16-wk experiment without clinical signs of poor health.

From the initiation to week 8 of this study, pigs were fed diets containing no MA. After consuming diets for 8 wk, pigs fed HP-DDG had reduced (*P* < 0.05) BW compared with those fed CON (Table 5). This decrease in BW was the result of less ADG of pigs consuming HP-DDG diets, where differences in ADG between CON and HP-DDG treatments accrued as time progressed (diet × period interaction; *P* < 0.05). The reduction of ADG and BW was the result of less ADFI in pigs fed HP-DDG diets than those fed CON diets (*P* < 0.05). However, gain efficiency during the first 8 wk was not different between the two dietary treatments. Recently, studies showed that adding corn coproducts to diets may lead to an imbalance of branched chain AA as a result of the high dietary Leu concentration, which can reduce feed intake and, ultimately, growth performance of pigs (Cemin et al., 2019; Kwon et al., 2019; Yang et al., 2019). However, diets containing HP-DDG in the current study contained not only high Leu concentrations but also adequate levels of Ile and Val based on recommendations from Htoo et al. (2017) and Wiltafsky et al. (2010). In addition, using a recently published prediction equation by Cemin et al. (2019), the predicted ADFI of pigs fed HP-DDG was similar to pigs fed CON. Therefore, the reduction of ADFI in Exp. 1 was not likely due to high Leu concentration in the HP-DDG diets. Because of the potential confounding effects of low levels of mycotoxin contamination of the HP-DDG source used in this experiment, it was unclear whether these differences in growth performance were a result of adding 30% HP-DDG to the diets (Widmer et al., 2008; Seabolt et al., 2010) or mycotoxin contamination (Patience et al., 2014; Frobose et al., 2015). Therefore, in an attempt to determine if the reduction in ADFI and ADG observed during the first 8 wk of this study was due to the addition of 30% HP-DDG to the diets or the low concentrations of mycotoxins in the HP-DDG diets, we chose to modify the experiment to a 2 × 2 factorial arrangement of treatments to consist of CON or HP-DDG diets with and without 0.25% MA for the subsequent 8-wk feeding period. The MA we chose to use has been reported to consistently ameliorate reductions in feed intake and growth rate caused by diets contaminated with DON in previous studies (Patience et al., 2014; Frobose et al., 2015; Thanh et al., 2015).

**Table 5.** Effects of feeding diets containing 30% HP-DDG contaminated with mycotoxins on growth performance during weeks 0–8 in Exp. 1

	Dietary treatment		
Item	CON	30% HP-DDG	SEM
BW, kg			
Week 0	20.32	20.31	0.75
Week 2	29.27^a^	27.55^b^	0.75
Week 4	40.75^a^	37.65^b^	0.75
Week 6	53.95^a^	49.79^b^	0.75
Week 8	67.23^a^	62.50^b^	0.75
Overall ADG, kg/d	0.83^a^	0.75 ^b^	0.01
Overall ADFI, kg/d	1.84^a^	1.69^b^	0.04
Overall G:F	0.46	0.45	0.03

^a,b^Means with different superscripts within a row are different (*P* < 0.05).

Because pigs fed HP-DDG diets had reduced BW at the end of week 8 compared with those fed CON, pig BW after treatment reassignment was used as a covariate in the analysis for BW, ADG, ADFI, and G:F during the final 9–16-wk feeding period. Pigs in all dietary groups had similar adjusted BW at the beginning of week 9 but, after the addition of 0.25% MA to diets, pigs fed HP-DDG + MA diets had greater final BW than pigs fed the HP-DDG diets without MA (Table 6). A diet × MA interaction was observed for ADFI (*P* = 0.01), which indicates that the addition of MA improved ADFI in pigs fed HP-DDG but reduced ADFI in pigs fed CON. The overall G:F ratio was greater (*P* = 0.02) in pigs fed either the CON or HP-DDG diets with MA than pigs fed these diets without MA. There was also a trend (*P* = 0.06) for a diet × MA interaction for ADG, which indicated that the addition of MA only improved ADG in pigs fed HP-DDG but not those fed CON. Therefore, the addition of MA to the HP-DDG diet was effective in mitigating the negative effects of mycotoxin contamination, which allowed pigs fed those diets to reach similar BW and achieve similar ADG and ADFI as those fed the CON diets. This observation is consistent with results reported by Patience et al. (2014), where adding MA reduced the negative effect of DON on ADFI and improved ADG in growing–finishing pigs. Shawk et al. (2019) observed a growth response of MA in nursery pigs regardless of DON levels and suggested that MA could lead to a growth response in addition to effective binding of mycotoxins. However, Shawk et al. (2018) did not observe similar effects in a finishing pig study. Results from the current study suggest that the reductions in ADFI and ADG observed during the first 8 wk of the study were due to additive effects of low concentrations of DON, FUM, and ZEA. However, it is unclear why the addition of MA reduced feed intake in pigs fed CON + MA.

**Table 6.** Effects of feeding mycotoxin-contaminated diets containing 30% HP-DDG diets with or without mycotoxin MA supplementation on pig growth performance from weeks 9–16 in Exp. 1^*a*^

	Treatment							
	CON		30% HP-DDG					
Item	−MA	+MA	−MA	+MA	SEM	Trt	MA	Trt × MA
BW, kg								
Weekk 9	64.88	64.92	64.57	64.64	0.86	0.77	0.95	0.99
Week 10	78.39^a^	78.94^a^	77.24^b^	79.66^a^	0.86	0.83	0.08	0.24
Week 12	92.18^ab^	92.17^ab^	90.56^b^	93.49^a^	0.86	0.89	0.09	0.12
Week 14	105.05^ab^	104.90^ab^	103.86^b^	106.54^a^	0.86	0.82	0.13	0.17
Week 16^*b*^	113.05^ab^	112.90^ab^	112.32^b^	115.05^a^	0.87	0.49	0.14	0.13
ADG, kg/d	0.93^ab^	0.92^ab^	0.90^b^	0.95^a^	0.02	0.86	0.18	0.06
ADFI, kg/d	2.80^a^	2.65^b^	2.64^b^	2.72^ab^	0.05	0.39	0.35	0.01
Overall G:F	0.33^b^	0.35^a^	0.34^ab^	0.35^a^	0.02	0.34	0.02	0.62

^*a*^Initial BW was used as a covariance in analysis.

^*b*^Pigs were harvested at day 3 of the last week (week 16).

^a,b^Means with different superscripts within a row are different (*P* < 0.05).

Recommendations for “safe” maximum concentrations of DON (<1 mg/kg), total aflatoxins (<200 µg/kg), ZEA (<1 mg/kg), and total FUM (5 mg/kg) in growing–finishing pig diets are commonly used as a guide to determine the relative risk of growth performance reductions when feeding mycotoxin-contaminated diets (Thaler and Reese, 2010). Etienne and Wache (2008) suggested that feed intake and growth rate of pigs would be expected to decrease only when more than 1 mg/kg DON were present in feed, and sustained feed intake reduction only occurs when more than 4 mg/kg DON is present in feed. However, these recommendations only apply to DON and do not account for the presence of other DON metabolites or other co-occurring mycotoxins that may be present. In the present study, the diet concentration of DON was low (0.7 mg/kg) and within the suggested safe levels reported by Thaler and Reese (2010) and Etienne and Wache (2008), but a reduction in ADFI was observed. Eriksen and Pettersson (2004) reviewed and summarized numerous feeding trials involving feeding various diet concentrations of DON to pigs and reported that decreased feed intake was observed when DON concentration of diets was as low as 0.35 mg/kg, but the lowest DON concentrations that have been shown to have negative effects on feed intake varied among studies. More recently, Frobose et al. (2016) observed inconsistent feed intake reductions based on DON concentrations in swine diets, where feeding a diet containing 1.7 mg/kg DON caused an 8% reduction in feed intake, but feeding a diet containing 3.2 mg/kg DON only led to a 2% decrease in ADFI compared with control diets. Therefore, the magnitude and inconsistency of feed intake and growth rate reductions observed among studies may be a result of the presence of other DON metabolites and derivatives (that were not measured) or synergistic effects with co-occurring mycotoxins (i.e., FUM and ZEA). Mycotoxin metabolites and derivatives are often described as “masked” mycotoxins, which cannot be detected using conventional chemical analysis techniques, and no safe limits in swine diets have been established, but these compounds can cause biological toxicity (Berthiller et al., 2013; Nagl et al., 2014; Pierron et al., 2016). In addition, the majority of grains are cocontaminated with a mixture of mycotoxins produced by the same mold (e.g., *Fusarium sp*; Grenier and Oswald, 2011). Very few studies have investigated interactive effects of co-occurring mycotoxins, but several researchers have suggested that combinations of multiple mycotoxins could have additive effects when contaminated feeds are fed to animals (Huff et al., 1988; Speijers and Speijers, 2004; Smith et al., 2016). Combinations of FUM and DON have been reported to have additive or synergistic effects on morphological and immunological changes (Bracarense et al., 2012) and growth performance (Grenier and Oswald, 2011). Therefore, the reduced ADFI and ADG observed in the current study may be attributable to additive effects of low concentrations of DON, FUM, and ZEA even though their individual concentrations are considered to be below recommended levels of concern.

### Exp. 2 Growth Performance

During the course of Exp. 2, three pigs (CON = 1 and HP-DDG = 2) were removed from the study for reasons unrelated to the dietary treatments. All other pigs remained on their respective dietary treatments for the entire 16-wk feeding period without visible signs of poor health. The HP-DDG source used in this experiment was produced using the same processes used for producing the HP-DDG source used in Exp. 1, but the HP-DDG used in Exp. 2 had greater FUM concentrations and less DON and ZEA content. Because of the ADFI and ADG reductions observed from feeding the HP-DDG source in Exp. 1 and the effectiveness of mitigating these reductions when MA was added during the last 8 wk of the feeding period, MA was added to CON and 30% HP-DDG diets at the beginning of this experiment to prevent reductions in growth performance from low concentrations of mycotoxins. An additional mycotoxin MA (Defusion 501; Provimi, Lewisburg, OH) was also added because of the greater FUM content from this HP-DDG source, which was recommended by the supplier of these products.

During the entire 16-wk feeding period, pigs fed the 30% HP-DDG diets had less final BW (*P* < 0.01) and ADG (*P* < 0.01) and tended (*P* = 0.06) to have lower G:F than pigs fed CON. However, there was no effect of diet on ADFI, and no interactions for diet × sex or diet × sex × period were observed for all growth performance measurements (Table 7). As expected, gilts had less final BW (*P* = 0.02), reduced ADG (*P* < 0.01) and ADFI (*P* < 0.01), but greater G:F (*P* = 0.02) than barrows, which is consistent with results from other studies (Chen et al., 1999; Wiseman et al., 2007; Xu et al., 2010). Widmer et al. (2008) reported no differences in ADG, ADFI, G:F, or BW for the entire growing–finishing feeding period when feeding diets containing up to 40% HP-DDG compared with feeding corn-soybean meal diets. Kim et al. (2009) observed no effects on overall ADG, ADFI, and G:F when 3–21% HP-DDG was used to replace soybean meal in growing–finishing pig diets. However, those previous studies used HP-DDG that was produced using old process technologies that are no longer available in the market today. Although there is no direct evidence in the current study, results of Exp. 1 suggest that the use of a commercial mycotoxin MA likely prevented a potential reduction in ADFI and ADG that may have occurred when feeding the 30% HP-DDG diets. The differences in final BW between dietary treatments in Exp. 2 resulted from cumulative reductions in ADG during the entire 16-wk feeding period, but this reduction was not a result of reduced feed intake because no differences in ADFI were observed between pigs fed HP-DDG and CON diets.

**Table 7.** Effects of feeding mycotoxin-contaminated diets containing 30% HP-DDG and MAs on growth performance (0–16 wk) in Exp. 2

	Diet		Sex			*P* value	
Item	CON	HP-DDG	Gilt	Barrow	SEM	Diet	Sex
BW, kg							
Week 0	22.75	22.74	22.68	22.81	1.20	0.99	0.91
Week 2	32.75	31.50	32.34	31.91	1.20	0.31	0.72
Week 4	45.95	43.91	44.83	45.02	1.20	0.11	0.88
Week 6	61.34	58.34	59.07	60.60	1.20	0.02	0.22
Week 8	77.19	73.07	73.65	76.61	1.20	<0.01	0.03
Week 10	93.54	87.50	88.70	92.35	1.20	<0.01	<0.01
Week 12	109.11	102.86	103.24	108.73	1.20	<0.01	<0.01
Week 14	123.89	116.94	117.51	123.32	1.20	<0.001	<0.01
Week 16	133.37	126.58	127.34	132.61	1.20	<0.001	<0.01
ADG, kg							
Weeks 0–2	0.72	0.63	0.69	0.65	0.04	0.03	0.30
Weeks 2–4	0.94	0.89	0.89	0.94	0.04	0.18	0.27
Weeks 4–6	1.10	1.03	1.01	1.11	0.04	0.11	0.02
Weeks 6–8	1.13	1.05	1.04	1.15	0.04	0.06	0.01
Weeks 8–10	1.17	1.03	1.08	1.12	0.04	<0.01	0.25
Weeks 10–12	1.11	1.08	1.04	1.16	0.04	0.48	<0.01
Weeks 12–14	1.06	1.01	1.02	1.04	0.04	0.22	0.54
Weeks 14–16	0.86	0.88	0.89	0.85	0.04	0.74	0.26
Overall	1.01	0.95	0.96	1.00	0.01	<0.01	<0.01
ADFI, kg							
Weeks 0–2	1.25	1.22	1.26	1.20	0.07	0.68	0.40
Weeks 2–4	1.81	1.80	1.82	1.79	0.07	0.84	0.71
Weeks 4–6	2.32	2.29	2.22	2.39	0.07	0.75	0.02
Weeks 6–8	2.71	2.64	2.50	2.85	0.07	0.26	<0.01
Weeks 8–10	3.07	2.94	2.79	3.22	0.07	0.07	<0.01
Weeks 10–12	3.30	3.16	3.00	3.46	0.07	0.05	<0.01
Weeks 12–14	3.35	3.21	3.16	3.41	0.07	0.05	<0.01
Weeks 14–16	3.25	3.33	3.21	3.36	0.07	0.27	0.04
Overall	2.63	2.57	2.49	2.71	0.04	0.16	<0.01
G:F							
Weeks 0–2	0.58	0.51	0.55	0.54	0.01	<0.01	0.46
Weeks 2–4	0.52	0.50	0.49	0.53	0.01	0.08	0.01
Weeks 4–6	0.47	0.45	0.46	0.47	0.01	0.11	0.52
Weeks 6–8	0.42	0.40	0.42	0.40	0.01	0.21	0.31
Weeks 8–10	0.38	0.35	0.38	0.35	0.01	0.06	0.01
Weeks 10–12	0.34	0.34	0.35	0.33	0.01	0.67	0.31
Weeks 12–14	0.32	0.31	0.32	0.30	0.01	0.73	0.14
Weeks 14–16	0.26	0.26	0.28	0.25	0.01	0.93	0.04
Overall	0.41	0.39	0.41	0.40	0.01	0.06	0.02

Formulating diets using the NRC (2012) ME value for HP-DDG seems appropriate based on the ADFI responses observed, which suggests that the reduction in ADG may have been due to the use of inaccurate SID AA estimates for this HP-DDG source when formulating these diets. Although diets were formulated to meet or exceed NRC (2012) AA requirements and the SID content of most AA appeared to exceed the requirements, if the digestibility for these AA was overestimated, it is possible that the pigs fed HP-DDG diets consumed inadequate amounts of AA to achieve optimal growth. In fact, a recent study conducted by Rho et al. (2017) evaluated the SID AA and ME content of HP-DDG produced using the same process technology as HP-DDG used in the current study. Two lots of HP-DDG used in that experiment were produced by the same ethanol plant but were collected at different time points. Although the chemical composition of the two HP-DDG samples was similar, SID coefficients of AA varied and were quite different from NRC (2012) values. For instance, SID of Lys was 47.2% in one HP-DDG sample and 55.9% in the other sample from the same ethanol plant, while NRC (2012) reported a SID Lys coefficient of 69%. Using the SID AA coefficients reported by Rho et al. (2017), we recalculated the SID AA content in our diets. Results from this theoretical comparison showed that the SID AA content in our HP-DDG diets were less than expected (Table 8). Depending on the dietary phase, specific AA, and digestibility coefficients used, the SID AA content was 1–13% less than calculated values in our initial diet formulations. Even though we used a 5% safety margin above NRC (2012) requirement when formulating diets, some AAs were still likely to be deficient. For instance, the recalculated SID Lys in phase 4 HP-DDG diet was 11% less than SID Lys in the initial formulation, which accounts for a 2.3 g/d difference (17.4 vs. 19.7 g/d SID Lys, respectively) in SID Lys intake, resulting in the diet being deficient in Lys compared with the NRC (2012) requirement of 17.9 g/d. Therefore, a reduction in ADG may have resulted from reduced SID AA intake caused by less than expected SID AA content in the actual diets. Further evidence of inadequate SID AA intake of pigs fed the HP-DDG diets was observed in carcass trait responses.

**Table 8.** Comparison of formulated SID AA content in diets (Exp. 2) based on NRC (2012) SID estimates for HP-DDG and recalculated diet SID AA content based on the values reported by Rho et al. (2017)^*a*^

	Phase 1			Phase 2			Phase 3			Phase 4		
Item	NRC	HP-A	HP-B	NRC	HP-A	HP-B	NRC	HP-A	HP-B	NRC	HP-A	HP-B
Lys, %	1.07	1.00	1.03	0.87	0.80	0.83	0.74	0.67	0.70	0.65	0.58	0.60
Thr, %	0.65	0.59	0.62	0.55	0.49	0.51	0.48	0.43	0.45	0.44	0.38	0.41
Met, %	0.45	0.43	0.44	0.42	0.39	0.41	0.40	0.37	0.39	0.38	0.36	0.37
Ile, %	0.75	0.69	0.73	0.63	0.57	0.61	0.55	0.49	0.53	0.50	0.44	0.48
Val, %	0.91	0.86	0.89	0.79	0.74	0.77	0.72	0.66	0.70	0.66	0.61	0.65
Arg, %	1.09	1.03	1.06	0.88	0.81	0.84	0.74	0.68	0.71	0.65	0.58	0.62
His, %	0.52	0.48	0.50	0.45	0.41	0.43	0.40	0.36	0.38	0.37	0.33	0.35
Leu, %	1.94	1.88	1.92	1.77	1.71	1.74	1.66	1.60	1.64	1.58	1.53	1.56
Phe, %	0.97	0.93	0.95	0.84	0.80	0.82	0.76	0.72	0.74	0.70	0.66	0.68

^*a*^SID AAs of high-protein distillers grains were calculated based on NRC (2012) published values for corn HP-DDG or two sets of SID coefficients (HP-A and HP-B) published by Rho et al. (2017). The HP-DDG (A and B) were produced at the same plant but different time points with similar composition.

### Exp. 2 Carcass Composition

Overall, pigs fed HP-DDG diets had reduced (*P* < 0.01) HCW, carcass yield, LMA, and FFL% compared with pigs fed CON, but BF depth was not affected by dietary treatment (Table 9). The reduced HCW observed in the current study is inconsistent with other previous studies that showed no differences in HCW when feeding diets containing up to 30% HP-DDG to growing–finishing pigs (Widmer et al., 2008; Kim et al., 2009; Gutierrez et al., 2014). This reduction in HCW was a result of reduced ADG for pigs fed HP-DDG diets in the current study, which was not observed in previous studies. Therefore, HCW was used as a covariate in statistical analysis for carcass traits to minimize the confounding effects of final BW of pigs in the current study. Carcass yield was also reduced (*P* < 0.01) in pigs fed 30% HP-DDG diets compared with those fed CON, which is consistent with the observation reported by Gutierrez et al. (2014). Widmer et al. (2008) found no differences in carcass yield after feeding HP-DDG diets. However, in the Widmer et al. (2008) study, the dietary inclusion rate of HP-DDG was decreased from 40% in the grower diet to 20% in the late finisher diet, whereas, in the study by Gutierrez et al. (2014), as well as the current study, diets containing 30% HP-DDG were fed throughout the entire growing–finishing period until slaughter. Increased concentrations of dietary fiber may reduce carcass yield due to gut fill and increased intestinal mass (Kass et al., 1980; Just, 1982). The HP-DDG source used in the current study contained 36% NDF, resulting in the HP-DDG diets having 70–80% more NDF than CON diets. This response is consistent with several other studies where feeding 30% DDGS diets, in which DDGS had similar NDF content compared with HP-DDG, reduced carcass yield (Whitney et al., 2006; Xu et al., 2010; Wu et al., 2016a). Backfat depth was not different between pigs fed HP-DDG and CON, which is in agreement with other studies that evaluated the addition of earlier HP-DDG sources to growing–finishing pig diets (Widmer et al., 2008; Kim et al., 2009). Stein and Shurson (2009) also reported that 14 out of 15 studies that evaluated feeding up to 30% DDGS to growing–finishing pigs had no effect on BF thickness. Backfat depth can be affected by energy density of diets fed to growing–finishing pigs (Smith et al., 1999; Apple et al., 2004), which suggests that the calculated energy density of the experimental diets were likely to be similar, and the ME content for HP-DDG used in diet formulation for this study was appropriate.

**Table 9.** Comparison of carcass characteristics of pigs fed CON and 30% HP-DDG diets in Exp. 2^*a*^

	CON		30% HP-DDG			*P* value		
Trait	Gilt	Barrow	Gilt	Barrow	SEM	Diet	Sex	Diet × sex
HCW, kg	98.34	98.16	96.07	95.86	1.68	<0.01	0.70	0.97
Carcass yield, %	75.55	75.33	74.04	73.78	0.61	<0.01	0.48	0.95
BF depth, mm	19.75	23.45	20.72	23.22	0.91	0.57	<0.01	0.17
LMA, cm	49.26	44.91	42.58	43.71	1.29	<0.01	<0.05	<0.01
Fat-free lean, %	52.40	49.38	50.10	49.04	0.47	<0.01	<0.01	<0.01

^*a*^HCW was used as covariate for carcass yield, BF depth, LMA, and fat-free lean percentage in the statistical analysis.

As expected, gilt carcasses had less HCW (*P* = 0.01), BF depth (*P* < 0.01), but greater LMA (*P* < 0.05), and carcass FFL% (*P* < 0.01) than barrows, which is in agreement with results reported by Cromwell et al. (1993). A diet × sex effect (*P* < 0.01) was observed for LMA and FFL%, where barrows fed HP-DDG had similar LMA and FFL% as barrows fed CON, while gilts fed HP-DDG had reduced LMA and FFL% than gilts fed CON. Cromwell et al. (1993) showed that gilts were more sensitive to dietary Lys concentration changes than barrows, where LMA and lean growth rate of barrows had smaller changes when dietary Lys concentration was reduced, but the same magnitude of change in dietary Lys concentration for gilts resulted in a more dramatic linear reduction in LMA and lean growth. Therefore, these carcass composition responses provide additional evidence suggesting that using the NRC (2012) estimates for SID AA content of the HP-DDG source fed in the current study may have been overestimated.

### Exp. 2 Pork Fat Quality

Pork fat quality is influenced by the amount of dietary lipid consumed and the fatty acid composition of dietary lipid (Wood et al., 2004). The source of HP-DDG used in this experiment contained about 10% EE, which is similar to some conventional DDGS sources and much greater than reported for previously produced HP-DDG sources (3.5%) in NRC (2012). Feeding diets containing increasing inclusion rates of DDGS increases the unsaturated fatty acid content of pork carcass fat depots and reduces pork fat firmness (Xu et al., 2010; Wu et al., 2016b). Corn oil present in DDGS and HP-DDG contains a high (58%) concentration of linoleic acid (C18:2; NRC, 2012), which is the primary contributor to undesirable soft pork fat (Wood et al., 2004). Feeding high amounts of dietary lipid decreases de novo synthesis of fatty acids and favors direct deposition of dietary lipid in adipose tissue (Farnworth and Kramer, 1987). Therefore, feeding the 30% HP-DDG diets in the current experiment resulted in dietary EE content greater than 5% compared with 2–3% EE content in CON diets and resulted in higher deposition of linoleic acid in BF of pigs fed 30% HP-DDG diets compared with those fed CON diets (Table 10).

**Table 10.** Comparison of fatty acid profile in BF of pigs fed CON and 30% HP-DDG diets in Exp. 2^*a*^

	Diet		Sex			*P* value		
Item	CON	HP-DDG	Gilts	Barrow	SEM	Diet	Sex	Diet × sex
C14:0	1.35	1.21	1.25	1.31	0.03	<0.01	0.05	0.19
C16:0	24.90	21.88	22.43	24.36	0.44	<0.01	<0.01	0.66
C16:1	2.44	1.76	2.06	2.13	0.11	<0.01	0.52	0.73
C17:0	0.35	0.36	0.36	0.34	0.03	0.71	0.33	0.57
C18:0	12.70	10.98	11.19	12.49	0.55	0.02	0.01	0.85
C18:1	40.24	37.15	38.60	38.78	0.68	<0.01	0.80	0.46
C18:2	10.86	19.85	16.87	13.84	0.75	<0.01	<0.01	0.29
C18:3	0.52	0.55	0.59	0.48	0.03	0.33	<0.01	0.72
C20:0	0.24	0.20	0.22	0.24	0.02	0.10	0.26	0.41
C20:1	0.80	0.70	0.74	0.76	0.05	0.06	0.52	0.82
C20:4	0.21	0.28	0.27	0.21	0.02	<0.01	<0.01	0.41
SFA^*b*^	39.56	34.65	35.46	38.75	0.87	<0.01	<0.01	0.77
MUFA^*c*^	43.50	39.61	41.42	41.69	0.73	<0.01	0.71	0.47
PUFA^*d*^	12.06	21.50	18.44	15.12	0.80	<0.01	<0.01	0.30
IV^*e*^	57.72	69.98	66.49	61.21	1.16	<0.01	<0.01	0.44

^*a*^Concentrations of fatty acids are expressed as grams of fatty acid/100 g fat, including myristic (C14:0), palmitic (C16:0), palmitoleic (C16:1), margaric (C:17:0), stearic (C18:0), oleic (C18:1), linoleic (C18:2), linolenic (C18:3), arachidic (C20:0), gadoleic (C20:1), and arachidonic (C20:4).

^*b*^Total saturated fatty acids = ([C8:0] + [C10:0] + [C12:0] + [C14:0] + [C16:0] + [C17:0] + [C18:0] +[C20:0] + [C22:0] + [C24:0]); brackets indicate concentration.

^*c*^Total monounsaturated fatty acids = ([C14:1] + [C16:1] + [C18:1 − 9c] + [C18:1 − 11c] + [C20:1] + [C24:1]).

^*d*^Total polyunsaturated fatty acids = ([C18:2n-6] + [C18:3n-3] + [C18:3n-6] + [C20:2] + [C20:4n-6]).

^*e*^Calculated iodine value = ([C16:1] × 0.95 + [C18:1] × 0.86 + [C18:2] × 1.732 + [C18:3] × 2.616 + [C20:1] × 0.785 + [C22:1] × 0.723; AOCS, 1998).

Concentrations of SFA and MUFA in BF of pigs fed HP-DDG were less (*P* < 0.01) than pigs fed CON, while PUFA content and IV were greater (*P* < 0.01; Table 10). As expected, BF from gilts had less SFA and greater PUFA content and IV than in barrow carcasses due to less BF depth (Zhang et al., 2009). No diet × sex interactions were observed for any of the fat quality measurements. Pigs fed HP-DDG had about twice as much linoleic acid (C18:2) in BF compared with pigs fed CON. Because linoleic acid accounted for more than 90% of PUFA, the dramatic difference in linoleic acid concentration between the HP-DDG and CON diets was also reflected in PUFA content of BF, which was at the expense of SFA and MUFA content. The high concentration of unsaturated fatty acids in BF of pigs fed the 30% HP-DDG diets led to an IV of 69.98, which is considered acceptable based on recommendations by the NPPC (2000), which suggest that BF IV be no greater than 70. Furthermore, Boyd et al. (1997) suggested that an IV less than 74 could be considered as an indicator of acceptable pork fat quality. Therefore, although feeding 30% HP-DDG diets elevated the IV in BF in the current study, pork fat quality would be considered acceptable based on these guidelines.

It was interesting to observe that growth performance was affected by the inclusion of 30% HP-DDG in Exp. 2 but not in Exp. 1. The differences in the results are likely caused by differences in nutritional composition and digestibility between the two sources of HP-DDG. Although the two sources of HP-DDG were produced from the same processes, differences in corn origin and operational efficiencies of ethanol plants may lead to differences in nutritional composition and digestibility among HP-DDG sources. Diet composition was similar in both experiments, but the HP-DDG used in Exp. 1 contained 10% more Lys than the HP-DDG source used in Exp. 2. Therefore, it continues to be a challenge of using accurate ME and SID AA values when formulating optimal swine grower–finisher diets.

In conclusion, low concentrations of mycotoxins in HP-DDG reduced feed intake and weight gain in growing–finishing pigs, but reduction in growth performance was alleviated when effective mycotoxin MAs were included in the diets. Feeding pigs diets containing 30% HP-DDG reduced weight gain, carcass yield, LMA, and fat-free lean percentage but yielded acceptable pork fat quality.

*Conflict of interest statement*. Authors declare no conflict of interest.
